# Accurate Hand Detection from Single-Color Images by Reconstructing Hand Appearances

**DOI:** 10.3390/s20010192

**Published:** 2019-12-29

**Authors:** Chi Xu, Wendi Cai, Yongbo Li, Jun Zhou, Longsheng Wei

**Affiliations:** 1 School of Automation, China University of Geosciences, Wuhan 430074, China; xuchi@cug.edu.cn (C.X.); jchowcug@gmail.com (J.Z.); weilongsheng@163.com (L.W.); 2 Hubei Key Laboratory of Advanced Control and Intelligent Automation for Complex Systems, Wuhan 430074, China

**Keywords:** hands detection, hand appearance reconstruction, convolutional neural networks, generative adversarial network, human–computer interaction

## Abstract

Hand detection is a crucial pre-processing procedure for many human hand related computer vision tasks, such as hand pose estimation, hand gesture recognition, human activity analysis, and so on. However, reliably detecting multiple hands from cluttering scenes remains to be a challenging task because of complex appearance diversities of dexterous human hands (e.g., different hand shapes, skin colors, illuminations, orientations, and scales, etc.) in color images. To tackle this problem, an accurate hand detection method is proposed to reliably detect multiple hands from a single color image using a hybrid detection/reconstruction convolutional neural networks (CNN) framework, in which regions of hands are detected and appearances of hands are reconstructed in parallel by sharing features extracted from a region proposal layer, and the proposed model is trained in an end-to-end manner. Furthermore, it is observed that the generative adversarial network (GAN) could further boost the detection performance by generating more realistic hand appearances. The experimental results show that the proposed approach outperforms the state-of-the-art on public challenging hand detection benchmarks.

## 1. Introduction

The human hand plays a very important role in communication when people interact with each other and with the environment in everyday life. The recognition of hand gestures and human activities is closely related to the locations and directions of human hands. Therefore, detecting human hand reliably [1] from single color images or videos which are captured from common image sensors plays an important role in many computer vision-related applications, such as human–computer interaction [2,3], human hand pose estimation [4,5,6], human gesture recognition [7,8], human activity analysis [9], and so on.

In the computer vision field, the pipeline of hand-related applications usually contains three steps: (1) hand detection, (2) hand pose estimation and (3) static gesture recognition or dynamic action recognition. The second step is optional because gesture/action recognition can be performed with or without hand pose estimation. About five years ago, hand pose estimation and action recognition were the most challenging steps (or bottlenecks) in the pipeline even in the constrained environment (normally only single hand and simple background in an image) from which the hand can be easily detected or assumed to be already cropped. Therefore, the hand-related community primarily focused on the second and third steps in the past. However, nowadays hand pose estimation and gesture/action recognition in the constrained environments are reaching the mature level, and hand-related applications in an unconstrained environment (complex background and the number of hands in an image is unknown) will be an important trend in the future. In this condition, hand detection in an unconstrained environment becomes a new bottleneck in the hand-related works. Thus, the high precision hand detection method will be a crucial step in the pipeline for hand-related applications in unconstrained environment. In this paper, we focus on the hand detection algorithm.

Traditional hand detection methods primarily utilize low-level image features such as skin color [10] and shape [11,12] for hand region detection. Nowadays, convolutional neural networks (CNN) based detection approaches [13,14,15,16,17,18,19] are proved to be more robust and accurate [20,21,22] due to the discriminative deep features learned. However, compared to common objects, human hands are highly articulated, appearing in various orientations, scales, shapes, skin colors, and sometimes partial occlusions, therefore reliably detecting multiple human hands from unconstrained cluttering scenes remains to be a challenging problem.

To tackle this problem, we propose an approach to accurately detect human hands from single color images by reconstructing the hand appearances. It can also be applied to video clips, as video clips can be considered as sequences of single images. The spirit of our approach is primarily oriented from multitask learning [23] which improves generalization of the network by learning tasks in parallel while using a shared representation. The quality of the hand detection task is closely related to the diversity of hand appearances in terms of hand shape, skin color, orientation, scale, and partial occlusion, etc. As a result, the shared information contained in the training signal of the hand appearance reconstruction task can be utilized as an inductive bias to improve the performance of the hand detection task.

**Figure 1:**
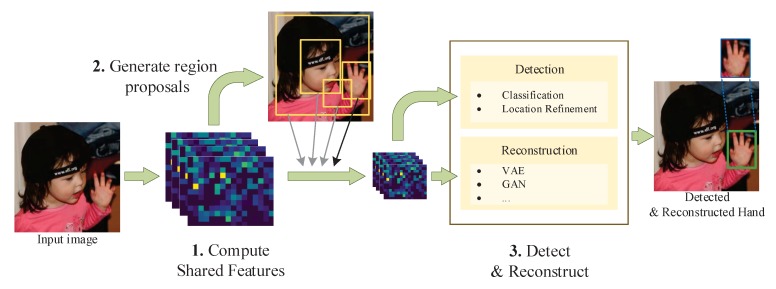
General process of our hybrid detection/reconstruction framework.

The general process of the proposed approach is illustrated in Figure 1. Firstly, feature maps of the whole input image are extracted by shared convolutional layers. Then, they are fed into a region proposal network (RPN) to generate possible region proposals, a.k.a. region of interest (RoI). Finally, the feature maps of RoIs are used to classify the corresponding labels (hand/background), to refine the locations of detected hands, and to reconstruct the appearances of hands simultaneously.

In this paper, a new hybrid detection/reconstruction CNN framework is present. The detection branch and reconstruction branch share the same feature presentation, so that the shared information contained in the hand appearance reconstruction branch can be utilized to boost the performance of the hand detection branch. We adopt the idea of variational autoencoder (VAE) [24,25] for hand appearance reconstruction. On the one hand, there is no previous work which adopted VAE in hand or object detection framework as far as we know. On the other hand, our approach is not a simple combination of the faster R-CNN, VAE, and GAN. Different from the traditional VAE model, our VAE model is asymmetric. During the detection, the shared information contained in the shared CNN and RPN layers can be effectively used by the asymmetric detection-VAE structure. Besides, it is found that GAN [26] could further improve the performance of the model by generating more realistic hand appearances.

To evaluate the proposed approach, we compare our approach with existing state-of-the-art methods on public hand detection benchmarks, i.e., Oxford hand dataset [27] and EgoHand dataset [28]. Experimental results show that our approach achieves the highest detection accuracy among the state-of-the-arts.

It is noted that there exist some related works which utilize generative methods for detection purpose as well, but their contributions are very different from ours. In [29] VAE is used to reconstruct traffic trajectories so that anomalies can be detected from videos. In [30] GAN is used to super-resolve small objects so that traffic signs can be detected reliably, and in [31] hard positive examples are generated by GAN to train a classifier which recognizes anomaly hard examples better. However, we aim at the challenging multiple hands detection problem which is very different from the detection tasks mentioned above. In our approach, the detection and reconstruction branches share the same features, and the detection performance is improved by introducing the shared information contained in the hand appearances reconstruction branch.

The rest of the paper is organized as follows. In Section 2, related works about hand detection and image reconstruction are reviewed. Details of the proposed approach are provided in Section 3. Experimental results are presented in Section 4. The details of our motivation and asymmetric VAE are discussed in Section 5. A brief conclusion is given in Section 6.

## 2. Related Works

Robust human hand detection in an unconstrained complex environment is one of the most challenging tasks in computer vision. The existing hand detection methods can be classified into two categories as follows.

*Traditional hand detection methods* use artificially designed weak features, such as skin color and shape features. These hand detection algorithms occupy less computational resources. In order to solve the skin color diversity problem caused by human races and illumination change, Girondel et al. [32] tried several color spaces and found that *Cb* and *Cr* channels in *YCbCr* color space worked well in skin detection task. Sigal et al. [10] proposed the Gaussian mixture model that performed well in different illumination conditions. But, the skin color based method is susceptible to the background that has similar color of skin. There are also some approaches that use shape features to detect hands. Guo et al. [12] trained a SVM classifier based on HOG features for detection. Based on [12], Mittal et al. [27] developed a mixture of deformable parts based method to detect hand precisely. Additionally, Karlinsky et al. [33] proposed an approach to locate hands by detecting the relative positions between the hands and other human body parts. However, because of the multi-scale and various rotations of human hands in a single picture, it is difficult to train a model suitable for unconstrained complex environment.

CNN-based detection methods are becoming a popular research topic in computer vision field recently, because higher-level deep features can be learned from the networks. The multi-scale and various rotations problems mentioned above can be well addressed by using CNN. Recent research has focused on three principal directions on developing better object detection systems, and these principals are also suitable for CNN-based hand detection. The three principal directions are introduced as follows.

The first principal direction is to change the base architecture of these networks. The main idea is adopting state-of-the-art convolutional networks to extract robust features, and it not only can benefit the classification but also detect location. Some recent work include ResNet [34], ResNet-Inception [35], and FPN [36] for object detection. Le et al. [21] proposed to fuse the multi-scale feature map directly for detection and classification to avoid missing small hands. Qing et al. [37] proposed a feature-map-fused SSD that used deconvolutional to combine deep layers with shallow layers for hand detection. However, the best detection precision in this direction is relatively low, i.e., 75.1% [21] on Oxford dataset. It is necessary to explore other directions to further improve the detection performance.

The second principal direction is to exploit the data itself by augmenting the diversity characteristics of the training data. Traditionally, the training data can be efficiently augmented by randomly generated spatial transformations [14,15], such as random translation, rotation, scaling and cropping, and it is a common technology widely used by the computer vision community. Besides, generative methods can also be utilized for data augmentation in hand-related applications. For example, in [38] GAN is adopted to augment training data for hand gesture recognition, and in [39,40] VAE and GAN are utilized to augment dataset for hand pose estimation. The generative methods can be employed to augment cropped image patches with single hand, but no previous work augments data using generative methods for hand detection task. The reason may be that, an unconstrainted scene usually contains multiple hands, and the contextual information among the hands and background is very hard to be modeled and randomized.

The third principal direction is to utilize contextual reasoning and proxy tasks for reasoning and other top-down mechanisms for improving representations of object detection. He et al. [16] proposed to use the segmentation as a way to contextually prime object detectors and provided feedback to initial layers. In [20,22,41], a hand rotation information was introduced to improve the detection precision. Deng et al. [20] adopted CNN to learn the rotation angles of hands, so that the directions of human hands were regulated by a spatial transformation network. Based on the idea of [20], Li et al. [22] proposed an embedded implementation of SSD (Single Shot Multi-Boxes) based hand detection and orientation estimation algorithm that can detect hand more efficiently. Narasimhaswamy et al. [41] introduced a contextual attention module for hand location and orientation estimation in unconstrained images. Most of the related works in this direction improve the representation for hand detection by utilizing the hand rotation information, and the best detection precision in this direction is 83.2% [22] on Oxford Dataset. However, we argue that the hand detection representation can be further improved by exploring more comprehensive information.

Our work follows this third principal direction. Hand appearances reconstruction is utilized to introduce shared information into our detection framework. Different from previous hand detection tasks, which only introduced rotation or orientation related information, reconstruction can deal with much more complex information of hand, such as scales, shapes, skin colors, and sometimes partial occlusions of hand.

To the best of our knowledge, there is no VAE based method for hand detection. VAE [24,42,43,44] can be used to reconstruct images effectively, but the VAE methods only minimized mean square error (MSE) between the reconstructed and original images, and the reconstructed image might look blurry. Therefore, we utilize GAN to improve the reconstructed image.

There are some related works that applying generative methods in detecting tasks, but their ideas are very different from ours. Kelathodi et al. [29] proposed a video object trajectory classification and anomaly detection method, in which VAE was used to reconstruct gradient conversion of trajectories and anomalies were detected by t-SNE. Li et al. [30] proposed perceptual generative adversarial networks by which small objects (e.g., traffic signs) were super-resolved to narrow the representation differences between objects of various scales. Wang et al. [31] utilized GAN to generate hard positive examples for training of a classifier which could recognize anomaly hard examples more accurately.

GAN based methods are also utilized in hand-related tasks such as abnormal dynamic gesture recognition [38] and gesture translation [45], but these works are very different from the image-based hand detection task which this work focuses on. For example, in [38] is addressed by dynamic hand gesture classification and abnormal hand gesture detection problemes using GAN, but their method was based on Electromyography (EMG) signals acquired from the forearm muscles (i.e., UC2018 DualMyo and UC2017 SG dataset). In [45] GAN was adopted to translate one hand gesture to another where only single hand appeared in per image. To the best of our knowledge, the GAN based method has not been used in hand detection applications previously.

## 3. Approach

**Figure 2:**
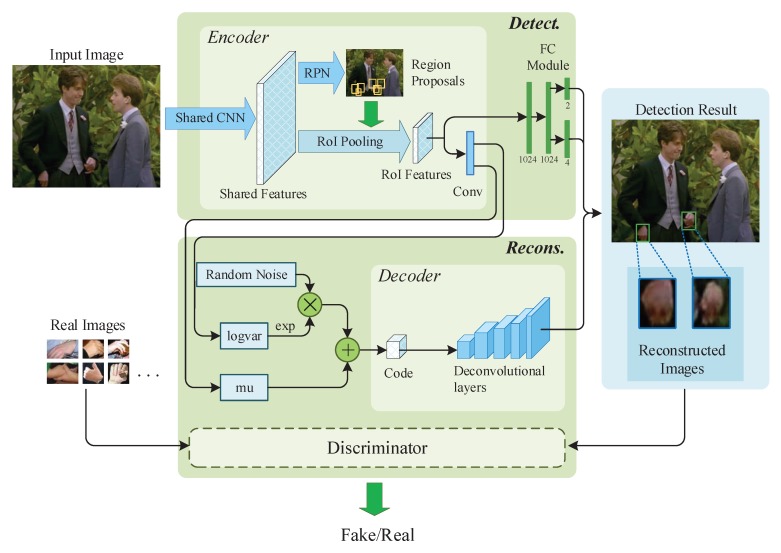
The framework of our model. The model consists of two branches, the detection branch and the reconstruction branch. Firstly, the input images are fed into shared convolutional neural networks (CNN) layers to compute shared features. Then, a region proposal network (RPN) is applied to generate region proposals, and pooling the feature of region proposal into the same shape. Finally, the region of interest (RoI) feature is used to detect hand and reconstruct hand appearance. Additionally, a discriminator is introduced to improve the reconstructed images.

In this work, we aim to train a model which can predict hand regions precisely and reconstruct the images of hand regions by using the RoI features. To achieve this, we present an end-to-end optimized detection and reconstruction framework, as shown in Figure 2. Our framework consists of two independent branches. One is the detection branch which is based on Faster R-CNN, and the other is the reconstruction branch which can reconstruct hand appearances in RoIs based on VAE. Both of these two branches share the same RoI features. Additionally, a GAN module is introduced to make the generated images look more realistic.

### 3.1. Detection Branch

In this part we adopt the backbone of the Faster R-CNN for hand detection. The modules of this branch can be divided into three parts, as follows.

The shared convolutional module computes shared features for region proposals generation, detection refinement and reconstruction. In general object detection network, VGG [46], ResNet [34,35] etc. are usually used as its shared convolutional layers. As can be seen in Figure 2, shared features are calculated by the shared convolution module, and then the features are used for generating region proposals, refining the bounding box location, and reconstructing hand appearances tasks. We augment the shared features of [16] by using feature pyramid network (FPN) [36]. The structure of FPN involves a bottom-up pathway, a top-down pathway, and lateral connections. The bottom-up pathway is the feedforward computation of the ResNet-101. The top-down pathway hallucinates higher resolution features by upsampling spatially coarser, but semantically stronger, feature maps from higher pyramid levels. Each lateral connection merges feature maps of the same spatial size from the bottom-up pathway and the top-down pathway.

The region proposal module generates region proposals. There are several methods, such as selective search [47], objectness [48] etc. However, these methods need to be trained independently, and are relatively inefficient. Therefore, we adopt RPN [15] which consists of 3×3 convolutional kernels to generate region proposals and can be integrated into an end-to-end training framework. Intersection-over-union (IoU) is usually employed to measure the overlap rate of two boxes, and the IoU is defined as

(1) $$\mathrm{IoU}=\frac{{\mathrm{Box}}_{1}\cap {\mathrm{Box}}_{2}}{{\mathrm{Box}}_{1}\cup {\mathrm{Box}}_{2}}.$$

For each generated region proposal, we consider it as positive if the IoU between region proposal and ground truth is larger than 0.7, and as negative if the IoU is smaller than 0.3. Then the non-maximal suppression (NMS) [49] method is used to remove region proposals with higher IoU overlap rate. In order to pool the region proposals with different scales and aspect ratios into same size (in this work, the size of RoI feature is 7×7), we generate a regular grid of size 14×14 evenly cover the region proposal for sampling purpose. As the region proposals are with different scales and aspect ratios, the x,y coordinates on the grid are float but not integer. A bilinear interpolation algorithm is used to compute the exact value of the shared feature on the grid, and then a max-pooling with stride of 2 is used to aggregate the feature to 7×7.

Classification and refinement modules are used to classify the regions and to refine the detected boxes. By utilizing fully connection layers (details refer to FC module of Figure 2), the RoI features are mapped to two vectors. One vector is softmax probabilities of background and hand (two-dimensional), the other vector is bounding-box regression offsets (four-dimensional).

### 3.2. Reconstruction Branch

**Figure 3:**
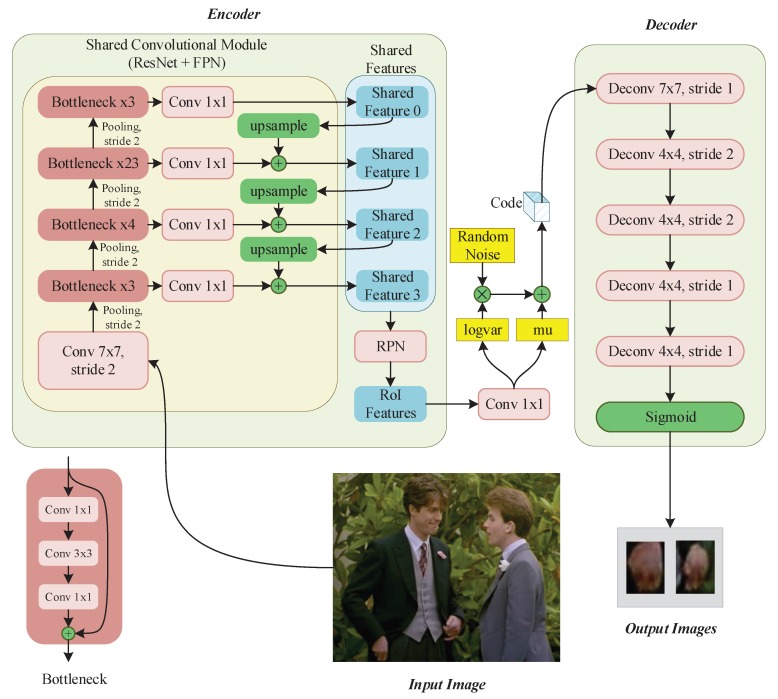
Asymmetric network structure of the encoder/decoder module.

**Figure 4:**
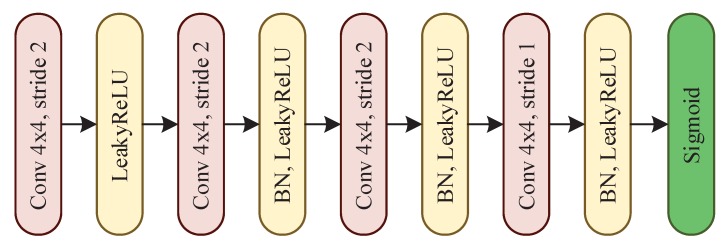
Network structure of the discriminator module.

In this part, we introduce a reconstruction branch (refer to Figure 3 and Figure 4) to reconstruct the hand appearances by using RoI features. Different from the traditional VAE model, in our model the structures of encoder and decoder are very different from each other (refer to Figure 3). Due to the input of our detection/reconstruction hybrid network is the whole image with arbitrary sizes, and the output of the reconstruction branch is image patches of uniform size (i.e., 32×32) that contain hands. To address the asymmetric of input and output, we design an asymmetric structure.

The encoder module is used to extract features of hand, in other words, encoding the image. It consists of two parts, shared convolutional module and Region proposal module which are described in the detection branch.

The decoder module is used to reconstruct hand appearances. Firstly, the RoI features are taken as the input of the convolutional layers with kernel size of 1×1 to calculate the mean vector μ and the logarithmic standard deviation vector σ of the RoI features. Then a Gaussian distributed noise Φ which has the same dimension of μ or σ is generated, and the latent vector c is calculated as follows

(2) $$c=\mu +\frac{e^{\sigma }}{2}\times \Phi .$$

This operation makes c follow a standard normal distribution roughly. Finally, c is fed into five deconvolutional layers and a sigmoid layer (refer to Figure 3) to generate hand appearance image.

The discriminator module discriminates whether the input image is fake or real. VAE based reconstruction model is trained by minimizing the element-wise error by default. The element-wise metric is simple but it does not model the properties of human visual perception, and the reconstruction of VAE tends to be blurry. An appealing property of GAN is that its discriminator network implicitly has to learn a rich similarity metric for images, so as to discriminate them from “unrealistic”. Thus, we introduce the discriminator into the reconstruction branch, which can make it learns more similarity features of hand appearance. By utilizing GAN, local details of the reconstructed hand tend to be finer and sharper. Because better local details contribute positively to the localization of hand, our approach with GAN demonstrates more accurate location prediction ability. In our scenario, the inputs of the discriminator module are the ground-truth hand patches cropped or reconstructed images outputted by the deconvolutional module. The discriminator module consists of four convolutional layers (refer to Figure 4). At the end of the module, a sigmoid function is applied to generate a probability which discriminates whether the input image is fake or real.

### 3.3. Loss Function

The loss of our proposed hybrid detection/reconstruction model is defined as

(3) $$L=L_{detection}+\lambda L_{recons},$$

where $L_{detection}$ is the loss of the detection branch, and $L_{recons}$ is the loss of the reconstruction branch.

For the detection branch, we mainly refer to a faster R-CNN and its loss is defined as

(4) $$L_{detection}=L_{rpn}+\gamma_{1}L_{detreg}+\gamma_{2}L_{cls},$$

where $L_{rpn}$ is the loss of RPN, $L_{detreg}$ is the loss of detection regression, and $L_{cls}$ is the loss of classification.

In the RPN module. At each pixel location on the shared features, we simultaneously predict *K* region proposals which are parameterized relative to *K* reference boxes, called anchors. Each anchor is centered at each pixel on the shared features, and is associated with scales and aspect ratios. Specifically, following [15], we use three scales and three aspect ratios, yielding K=9 anchors at each pixel. For regression of region proposal boxes, we adopt parameterizations of four coordinates as follows:

(5) $$t_{x}=\frac{x-x_{a}}{w_{a}},\;t_{y}=\frac{y-y_{a}}{h_{a}},\;t_{w}=\log\frac{w}{w_{a}},\;t_{h}=\log\frac{h}{h_{a}},$$

(6) $$t_{x}^{*}=\frac{x^{*}-x_{a}}{w_{a}},\;t_{y}^{*}=\frac{y^{*}-y_{a}}{h_{a}},\;t_{w}^{*}=\log\frac{w^{*}}{w_{a}},\;t_{h}^{*}=\log\frac{h^{*}}{h_{a}},$$

where *x*, *y*, *w* and *h* denote the two coordinates of the box center, width, and height. Variables $x^{*}$, $x_{a}$ and *x* are for the predict box, anchor box, and ground truth box respectively (likewise for *y*, *w*, *h*).

The anchor is matched with ground-truth firstly. It is counted as positive when IoU between the anchor and the ground-truth is larger than 0.7, and negative when the IoU is smaller than 0.3. For the positive anchor we set its label *l* as 1, and 0 for negative anchor. $L_{rpn}$ contains two parts, proposal regression loss $L_{propreg}$ and object score loss $L_{obj}$.

The proposal regression loss $L_{propreg}$ represents the relative distance between the region proposal and ground-truth, and it is calculated as follow

(7) $$L_{propreg}\left(t,t^{*}\right)=\frac{1}{N_{D}}\sum \left\{\begin{array}{cc} 0.5|t-t^{*}{|}^{2}, & \mathrm{if}|t-t^{*}|<1\\ |t-t^{*}|-0.5, & \mathrm{otherwise} \end{array}\right.,$$

where t is the parameter of the ground truth relative to the anchor offset which is calculated by using Equation (5). $t^{*}$ is the hypotheses for region proposals relative to the anchor offset corresponded to t. $N_{D}$ is the number of positive region proposals.

The object score loss $L_{obj}$ is log loss over two classes (background or object), it is defined as follow

(8) $$L_{obj}\left(l,l^{*}\right)=-\frac{1}{M_{D}}\sum \left[l^{*}\log\left(l\right)+\left(1-l^{*}\right)\cdot \log\left(1-l\right)\right],$$

where l is the label vector of anchors. $l^{*}$ are the hypotheses for region proposals label vector corresponded to l, and $M_{D}$ is the sum of negative and positive region proposals.

Due to the numbers of the human hand in different images are different, so for each image, $N_{D}$ and $M_{D}$ may be different. The regression loss of bounding-box refinement and classification loss are calculated similar to $L_{propreg}$ and $L_{obj}$ respectively.

For the reconstruction branch, we calculate the MSE loss between the real image and the reconstructed image as

(9) $$L_{recons}\left(P,P^{*}\right)=\frac{1}{N_{R}}\sum {\left|P-P^{*}\right|}^{2},$$

where P is the reconstructed image patch of cropped hand region, and $P^{*}$ is real image patch of ground-truth. $N_{R}$ is the number of reconstructed images.

Additionally, we also introduce the discriminator of GAN into our framework to further improve the performance. In this part, we need to distinguish whether the images which are fed into the discriminator are real or fake, so we adopt the cross-entropy loss function as

(10) $$L\left(p,p^{*}\right)=-\frac{1}{M_{R}}\sum \left[p^{*}\log\left(p\right)+\left(1-p^{*}\right)\cdot \log\left(1-p\right)\right],$$

where $p^{*}$ is the label vector of input images (the real image is labeled as 1, and the fake as 0), and p is the probability vector output by the discriminator. $M_{R}$ is the number of input images. During the training, the loss of GAN is calculated as follows

(11) $$L_{D}=-\frac{1}{M_{R_{1}}}\sum \left(\log\left(D\left(\mathrm{real}\right)\right)+\log\left(1-D\left(\mathrm{fake}\right)\right)\right),$$

(12) $$L_{G}=-\frac{1}{M_{R_{2}}}\sum \log\left(D\left(G\left(r\right)\right)\right),$$

where, *D* is the discriminator, *G* is the re-constructor, real is the real image of ground truth, fake is the reconstructed image, r is the feature output by region proposal network, $M_{R_{1}}$ is the number of all input images which include the real and reconstructed images, and $M_{R_{2}}$ is the number of reconstructed images. The training of GAN is split up into two main steps. In the first step, we train the discriminator to maximize the probability of correctly classifying a given input as real or fake. We assign the label of real image cropped on original image as 1 and the label of fake image generated by the reconstruction branch as 0. Then the real and fake images are fed into the discriminator to minimize the loss of discriminator $L_{D}$, and only the weights of discriminator are updated. In the second step, we minimize the loss $L_{G}$ by updating the weights of the whole network except the discriminator.

## 4. Experiments

In the following subsections, experiments will be conducted on the Oxford Hand Dataset and EgoHand Dataset to evaluate the effectiveness of our proposed approach. Our approach has two variations: (1) ours without GAN denotes to detect hand and reconstruct hand appearances without GAN; (2) ours with GAN denotes to detect hand and reconstruct hand appearances with GAN.

For each experiment, training samples are augmented by image processing operations such as resizing, shifting, flipping images, etc. which can further increase the number of training data, and prevent the over-fitting. In terms of training model, we adopt stochastic gradient descent (SGD) algorithm with a weight decay of 0.0005 and a momentum of 0.9 to update the weight of the model. The initial learning rate is 0.005, and then it will be multiplied by 0.1 per 3 epoch. The total training epoch is 10. All experiments are performed on a workstation with an Intel iCore 7 CPU, 32G RAM and a GTX 1080Ti GPU with 11G Memory capacity, and the program of our proposed approach is developed under the PyTorch deep learning framework.

### 4.1. Oxford Hand Dataset

This dataset [27] is a comprehensive images dataset of hands which are collected from various different public image dataset sources. In each image, all the hand instances that can be perceived clearly by humans are annotated. For the whole dataset, there are 13,050 hand instances. In the training set, there are 11,019 hand instances, and for the testing set, there are 2031 hand instances.

On the Oxford Hand Dataset, the proposed approach is compared with [13,15,16,20,21,22,27,41]. We adopt the evaluation metric proposed in [20], which uses the typical average precision when the threshold of IoU is 0.5 (${\mathrm{AP}}_{50}$).

**Figure 5:**
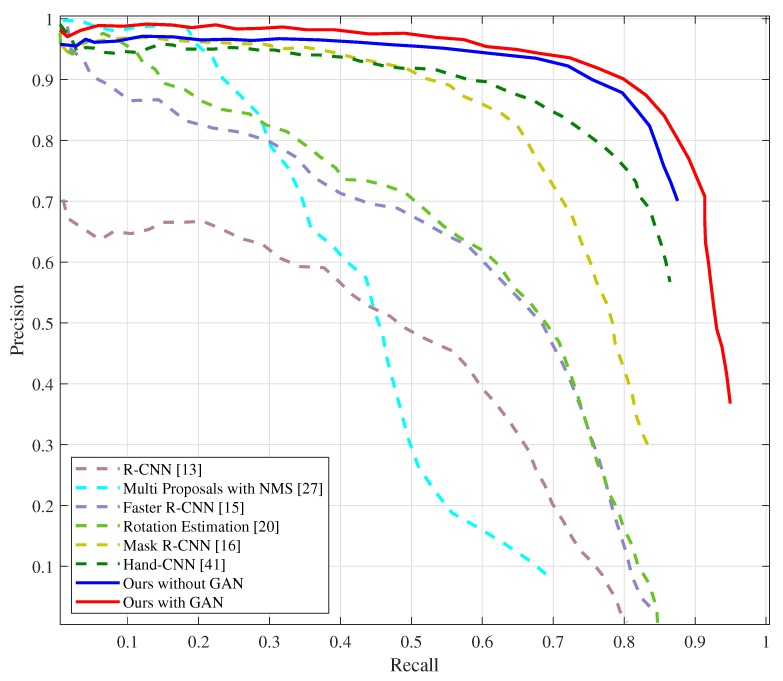
Precision-recall curves on the Oxford Hand Dataset. The curves of MS-FRCNN (Multiple Scale Region-based Fully Convolutional Networks) and SSD-hand are not shown because they are not provided in [21,22] (This figure is best viewed in color).

The ${\mathrm{AP}}_{50}$ of the state-of-the-art methods on the Oxford Hand Dataset are illustrated in Table 1, and their corresponding precision-recall curves are shown in Figure 5. We compared the results of our approach on the Oxford Hand Dataset with that of the state-of-the-art methods. When the threshold of IoU is 0.5, the average precision of our proposed approach without GAN (${\mathrm{AP}}_{50}$ = 87.0%) is higher than that of other methods. Our proposed approach with GAN (${\mathrm{AP}}_{50}$ = 87.6%) improves the average precision of the state-of-the-art method proposed in [22] by 4.4 points.

To further investigate the performance of our proposed approach, we conducted several experiments by using following variations: (1) baseline 1 [16] is the backbone of our approach, which to detect hand only without reconstruction, (2) baseline 2 augments *Baseline 1* by utilizing multi-scale feature maps [36], (3) ours without GAN and (4) ours with GAN mentioned above. We employ recently widely used evaluation metric [50], which includes the mean average precision at the threshold of IoU in the range from 0.5 to 0.95 (mAP), average precisions when the thresholds of IoU are 0.5 and 0.75 respectively (${\mathrm{AP}}_{50}$ and ${\mathrm{AP}}_{75}$). The evaluation results are shown in Table 2. The mean average precision of our model without GAN (mAP = 44.0%) improves 8.8% than the baseline 2 (mAP = 35.2%). It indicates that to reconstruct the hand appearances is helpful to improve the detection performance. As for the GAN, ${\mathrm{AP}}_{75}$ of our method with GAN is 42.0% which is 4.1 points higher than ours without GAN (${\mathrm{AP}}_{75}$ = 37.9%). It indicates that the GAN could improve the IoU between ground truth and predicted bounding-box. In other words, location prediction ability is improved by the GAN. Furthermore, the mAP of our method with GAN is 46.2% which is 2.2 point higher than ours without GAN (mAP = 44.0%). It shows that the comprehensive detection capability of ours with GAN is better than ours without GAN.

**Figure 6:**
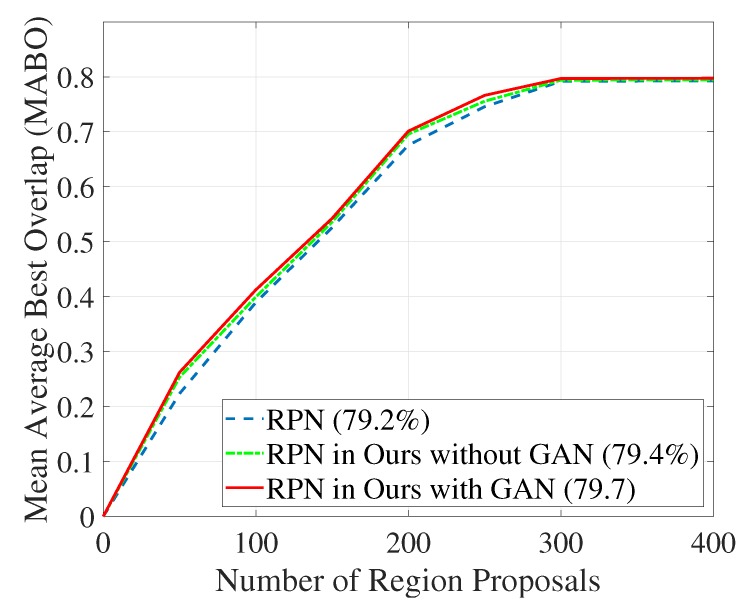
Trade-off between mean average best overlap and the number of region proposals.

In [20], the authors also measured the localization precision by using mean average best overlap (MABO) proposed in [47] to evaluate the localization ability of RPN, and determine the number of region proposals generated by RPN. Because the number region proposals is closely related to the efficiency of the network, in other words, the more region proposals generated, the more computing resources taken. In our work, we adopt the same RPN structure which is proposed in [14]. As shown in Figure 6, the MABOs of RPN in “ours without GAN” and “ours with GAN” converge to 79.4% and 79.7% respectively when the number of windows (region proposals) reaches 300. The RPN in [20], and its MABO converges to 79.2% when the number of windows reaches 300. Note that, [20] proposed a new RPN named “deep feature proposal” which achieves 100% recall when the number of windows is 17,489 (please refer to Table 3), but its MABO converges to 76.1% which is lower than that of the standard RPN. Comparing with the standard RPN, the recall of RPN in our approach is also improved.

**Figure 7:**
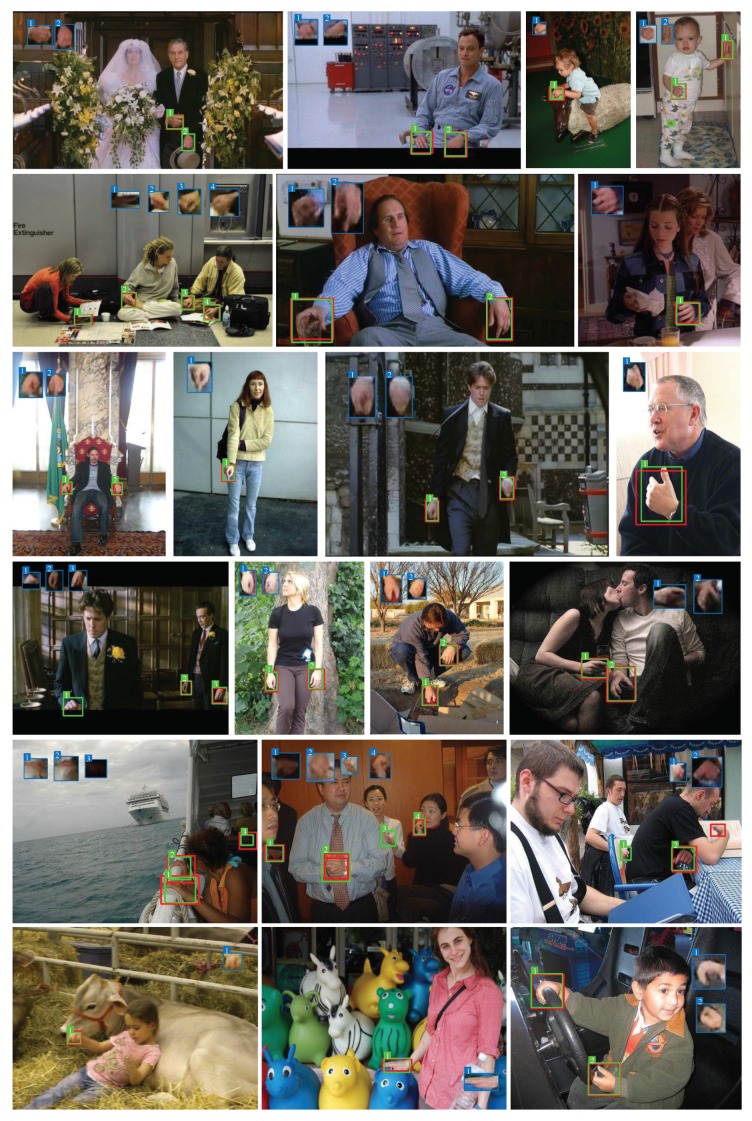
The detection results of our approach (ours with GAN) on the Oxford Hand Dataset. The red boxes denote the ground-truth of hands; the green boxes denote the predicted boxes output by our model; the blue boxes denote the reconstructed images of corresponding hand regions. Multiple hands are annotated with serial numbers on top left corners of boxes. (This figure is best viewed in color).

**Figure 8:**
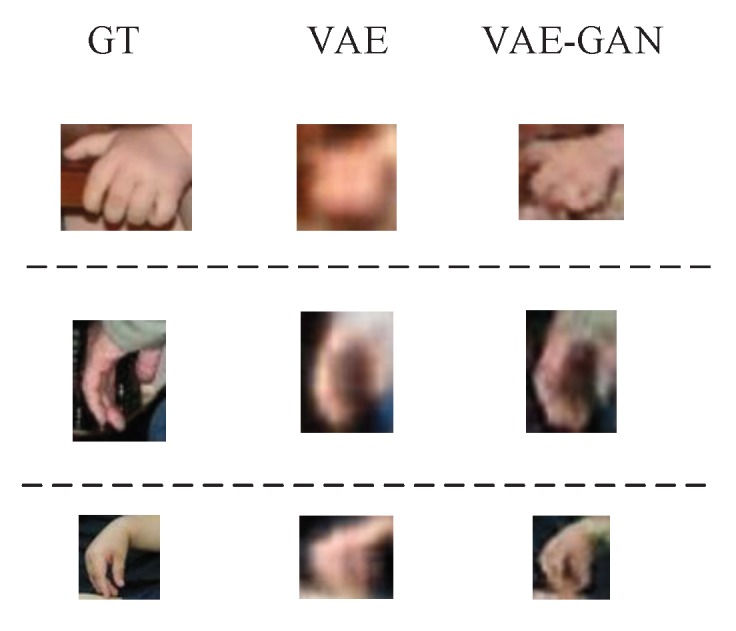
Comparison of reconstructed hand appearances between ours without GAN and ours with GAN. Each row corresponds to a sample. The first column denotes the ground truth, the second column denotes reconstructed images by ours without GAN, and the third column denotes reconstructed images by Ours with GAN.

**Figure 9:**
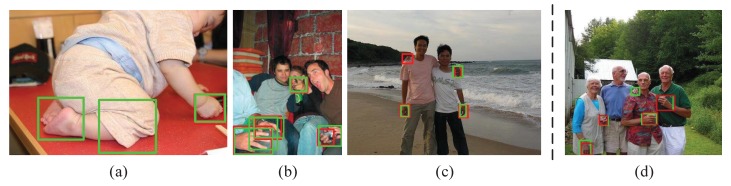
The failure case of our detection approach on the Oxford Hand Dataset. The red boxes denote the ground-truth of hands; the green boxes denote the predicted boxes output by our model. (This figure is best viewed in color).

Figure 7 shows some detection examples on Oxford Hand Dataset. Meanwhile, we also present the reconstructed images of hand appearances which are demonstrated in the blue boxes. Besides, from Figure 8, we can see that reconstructed images generated by VAE without GAN are blurry. By utilizing GAN, local details of the reconstructed hand tend to be finer and sharper. Although our model can precisely detect hands, there are still some false detections or missing detections. For example, in Figure 9a, the appearance of the baby’s foot is very similar to that of human hand in terms of color and shape, so there exists a certain probability that a human foot would be falsely detected as a human hand. As can be seen in the bottom right corner of Figure 9b, when two hands are spatially close to each other, they may be falsely detected as a single hand. In the top left corner of Figure 9c, when the human hand is heavily occluded, the scale is too small and the appearance is too dark, it may cause missing detection. Additionally, “false detection” may be caused by false labels in dataset. For example, in the center of Figure 9d, correctly detected hand maybe considered as a “false” detection due to the mislabel of this hand in the dataset.

The running time of our approach is compared with that of the previous state-of-the-art methods [15,20,21,22,27] in Table 4. Our approach is very efficient, because it takes about 0.1 s per image. Ours spends less time than most of the state-of-the-arts except [15,22], because we introduce the additional reconstruction branch which requires slightly more computational time but improves the detection precision significantly. In these works, almost all computing is done by GPU except [27] and our approach is primarily compared with the GPU based method. In our works, our GPU is GTX 1080 Ti with 3584 CUDA core, and 11 GB memory capacity, and the other works used TITAN X with 3072 CUDA core and 12GB memory capacity. Since we do not have a TITAN X GPU, we cannot test our model on it. Assuming that the computing speed is proportional to the number of CUDA cores, the speed of ours could be about 0.1297 s/frame on TITAN X GPU. Thus, ours is faster than [20,21], but slower than [15] and [22]. In [21], it uses a more complex feature extraction network which slows down its detection speed. In [20], due to the rotation estimation and the derotation modules, it is less efficient than ours. What is more, our approach is almost real-time.

### 4.2. EgoHand Dataset

This dataset [28] contains 48 Google Glass videos of complex and first-person interactions, such as, playing cards, chess, puzzle and Jenga, between two people in different locations. There are 130,000 frames of videos in total, of which 4800 frames have been labeled, and 15,053 hand instances have been annotated.

**Figure 10:**
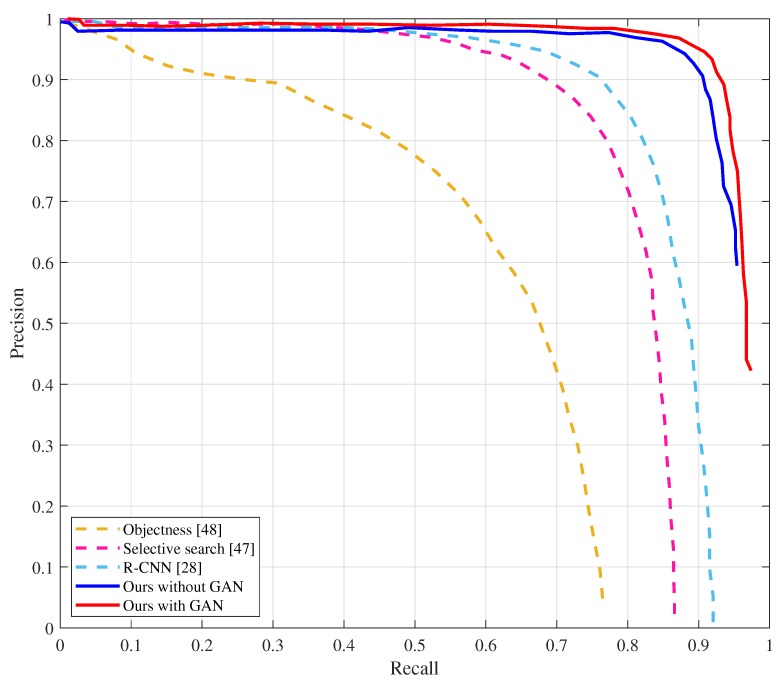
Precision-recall curves on the EgoHand Dataset. (This figure is best viewed in color).

**Figure 11:**
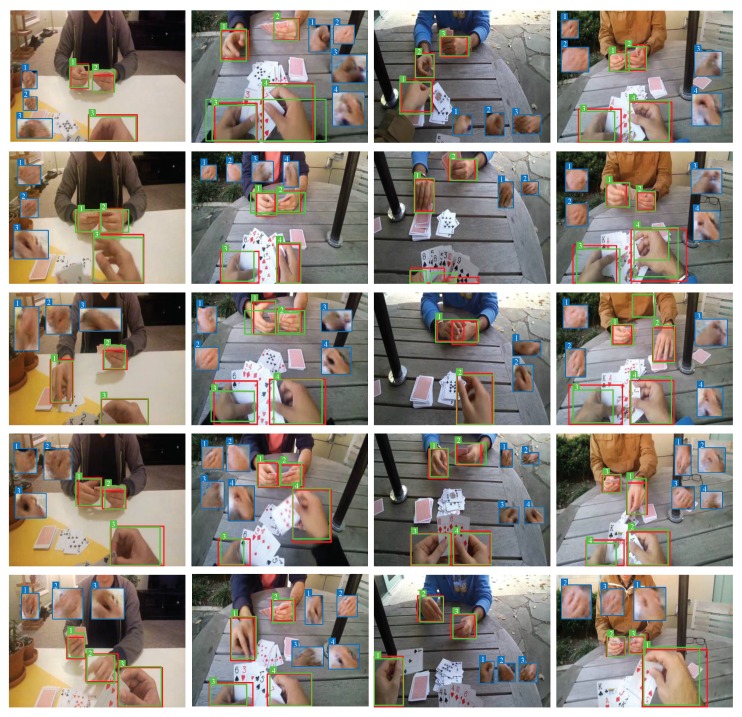
The detection results of our approach (ours with GAN) on the Egohand Dataset. The red boxes denote the ground-truth of hands; the green boxes denote the predicted boxes output by our model; the blue boxes denote the reconstructed images of corresponding hand regions. Multiple hands are annotated with serial numbers on top left corners of boxes. However, there are still some samples in which hands cannot be detected properly. For example, when two hands are very close with each other, the method would detect them as one hand. (This figure is best viewed in color).

In order to prove the hand detection ability of our approaches proposed in this work, we also evaluate our model on the EgoHand Dataset by calculating the average precision when the threshold of IoU is 0.5. The precision-recall curves are shown in the Figure 10, and the corresponding ${\mathrm{AP}}_{50}$ is illustrated in Table 5. The average precision of our proposed approach with GAN improves the highest score in literature by 10 points when the threshold of IoU is 0.5. Figure 11 shows some detection examples on Egohand Dataset. Besides, we also present the reconstructed images of hand regions which are demonstrated in the blue boxes.

In additionally, we also refer to the training method that is used in [37]. First, the model was pre-trained using the Oxford dataset. Then the EgoHand dataset is used to fine-tune the model. The model is evaluated by using evaluation metric proposed in [50]. The experimental results are shown in Table 6. It can be clearly seen that the pre-training and fine-tuning training method improves the performance of the model on the EgoHand dataset. The ${\mathrm{AP}}_{75}$ of ours with GAN improved 2.3% points.

**Figure 12:**
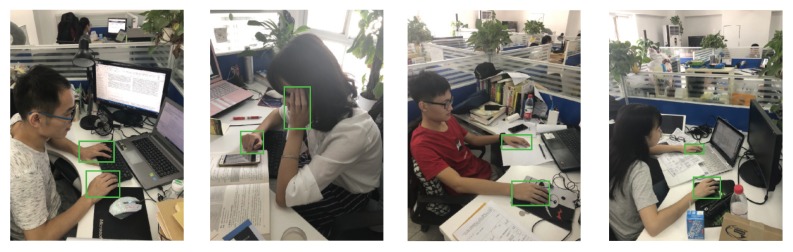
The detection results of our approach in the unconstrained real-life environment. The green boxes denote the predicted boxes output by our model. (This figure is best viewed in color).

Furthermore, to evaluate the performance of our approach in real-life environment, we test the model on color images captured by an off-the-shelf digital camera. As can be seen in Figure 12, our approach can reliably and precisely detect multiple human hands in unconstrained real-life environment.

## 5. Discussion

### 5.1. Motivation of Our Approach

Reliably detecting multiple hands from cluttering scenes is a challenging task because of complex appearance diversities of dexterous human hands in color images, for example, different hand shapes, highly articulated hand poses, skin colors, illuminations, orientations and scales etc. It is impossible to collect training samples evenly cover all these types of hand appearance diversities, and false labels are inevitable. To better understand the motivation of our approach, the details are discussed in the following aspects.

Detection without reconstruction: in this case, the optimizer pursuits merely minimizing the detection loss, and the network learns a model which best fits a specific training dataset. As human hand appearances are very complex in unconstrainted scenarios, the unbalance distribution of training samples, the bias, and false labels of specific training datasets would lead to overfitting and performance degeneration. Detection–only scheme may ignore important hand appearance features which could help generalization.

Detection and reconstruction with VAE: in this case, the optimizer pursuits not only maximizing the detection score, but also reconstructing the hand appearances as much as possible. The learned RoI features contain specific information for hand detection task, as well as comprehensive information for hand appearance reconstruction task. The detection/reconstruction scheme encourages the detector to classify labels and to regress locations based on learned RoI features with comprehensive hand appearance information, but not merely based on features for detection task only. Furthermore, the VAE enforces the learned RoI features (or to say “code”) to a normalized gaussian distribution, and this characteristic helps to alleviate the bias and overfitting problem.

Detection and reconstruction with VAE and GAN: an appealing property of GAN is that its discriminator network implicitly learns a rich similarity metric for appearance construction. By utilizing GAN, local details of the reconstructed hand tend to be finer and sharper. As better local details contribute positively to the localization of hand, our approach with GAN demonstrates more accurate location prediction ability.

### 5.2. Asymmetric VAE

The asymmetry of our VAE contains two aspects: (1) The input and output are asymmetric. (2) The structure of encoder/decoder is asymmetric.

The input and output are asymmetric: In traditional VAE models, one input image corresponds to one output image with the same size, and the contents of the images are the same. Whereas, in our VAE model, one input image corresponds to multiple output images. The input is the whole image with arbitrary size, and the outputs are image patches of uniform size (i.e., 32 × 32). The content of the input is unconstrained scenario (multiple hands and complex background), and the content of each output is the hand appearance corresponding to each detected region. Our VAE network is different from applying traditional symmetric VAE on the input image with a sliding-window fashion. In sliding-window-based methods, the regions are scanned evenly with constant step. But in our model, the region proposals are generated unevenly, and ratios of the region proposals vary from each other. The proposed method is different from applying traditional symmetric VAE on image patches cropped from detected regions. We do not extract features from cropped image patches, but based on the shared features, a bilinear interpolation algorithm is used to aggregate the features in the region proposal.

The structure of the encoder/decoder is asymmetric: the structure of the encoder and that of the decoder are very different from each other, which is asymmetric too. The encoder contains a shared convolutional module and a region proposal module. Whereas, the decoder only contains five deconvolutional layers and a sigmoid layer (refer to Figure 3 decoder module). For details, refer to Section 3.1 and Section 3.2.

## 6. Conclusions

In this paper we study the hand detection in unconstrained cluttering environment which is a challenging task because of complex appearance diversities of human hands. We propose a new hybrid detection/reconstruction convolutional neural network to reliably detect multiple hands as well as to reconstruct hand appearances. The main contribution of this paper is to improve the precision of hand detection by introducing the hand appearance reconstruction branch. What’s more, the detection precision can be further improved by introducing GAN to generate more realistic hand patches. The proposed approach is evaluated and analyzed on two widely-used hand detection benchmarks, i.e., Oxford Hand and EgoHand Datasets. Extensive experiments demonstrate the superiority of our hybrid detection/reconstruction framework for hand detection.

Although our proposed hybrid detection/reconstruction convolutional neural network can effectively detect human hand from unconstrained environments with high average precision, there are also some defects as illustrated in Figure 9. Besides it cannot distinguish right hand from left hand. So, it is important to deal with these issues in the future.

## Figures and Tables

**Table 1:** Average precision on the Oxford Hand Dataset when the threshold of IoU is 0.5.

Model	${\mathrm{AP}}_{50}$
R-CNN [13]	42.3
Multi Proposals with NMS [27]	48.2
Faster R-CNN [15]	55.7
Rotation Estimation [20]	58.1
Mask R-CNN [16]	70.5
MS-RFCN [21]	75.1
Hand-CNN [41]	78.8
SSD-Hand [22]	83.2
Ours without GAN	87.0
Ours with GAN	**87.6**

**Table 2:** Further comparison between our proposed approach and baseline.

Model	mAP	${\mathrm{AP}}_{50}$	${\mathrm{AP}}_{75}$
Baseline 1	31.5	70.5	22.1
Baseline 2	35.2	78.1	25.3
Ours without GAN	44.0	87.0	37.9
Ours with GAN	**46.2**	**87.6**	**42.0**

**Table 3:** Region proposals network performance on the Oxford Hand test data.

Model	Recall	MABO	#Proposals
Selective Search [47]	46.1%	41.9%	13,771
Objectness [48]	90.2%	61.6%	13,000
Deep Feature Proposal [20]	100%	76.1%	17,489
RPN [20]	95.9%	79.2%	300
RPN in Ours without GAN	96.8%	79.4%	300
RPN in Ours with GAN	98.7%	79.7%	300

**Table 4:** Average time to detect hands in the testing stage.

Model	Time	Test Environment	Framework
Multi proposals with NMS [27]	120 s	2.50 GHz CPU	Caffe
Faster R-CNN [15]	0.08 s	2.9 GHz CPU and Titan X	Caffe
RPN [20]	0.1 s	2.9 GHz CPU and Titan X	Caffe
Rotation estimation [20]	1.0 s	2.9 GHz CPU and Titan X	Caffe
MS-RFCN [21]	0.2150 s	3.5 GHz CPU and Titan X	Caffe
SSD-Hand [22]	0.0072 s	3.0 GHz CPU and Titan X	PyTorch
Ours without GAN	0.1112 s	3.5 GHz CPU and GTX1080Ti	PyTorch
Ours with GAN	0.1121 s	3.5 GHz CPU and GTX1080Ti	PyTorch

**Table 5:** Average precision on the EgoHand Hand Dataset when the threshold of IoU is 0.5.

Model	${\mathrm{AP}}_{50}$
Objectness [48]	56.8
Selective search [47]	72.9
R-CNN [28]	84.2
Ours without GAN	93.3
Ours with GAN	**94.2**

**Table 6:** Comparison between different datasets.

Dataset	Model	mAP	${\mathrm{AP}}_{50}$	${\mathrm{AP}}_{75}$
EgoHand	Ours without GAN	58.4	93.3	68.3
EgoHand	Ours with GAN	60.2	94.2	70.4
Oxford + EgoHand	Ours without GAN	59.1	93.6	69.0
Oxford + EgoHand	Ours with GAN	61.9	94.4	72.7
